# Periodontitis Salivary Microbiota Aggravates Ischemic Stroke Through IL-17A

**DOI:** 10.3389/fnins.2022.876582

**Published:** 2022-05-19

**Authors:** Yan-Lin Chen, Lan Bai, Dilirebati Dilimulati, Shuai Shao, Che Qiu, Ting Liu, Shuo Xu, Xue-Bing Bai, Lin-Juan Du, Lu-Jun Zhou, Wen-Zhen Lin, Xiao-Qian Meng, Yi-Chao Jin, Yan Liu, Xiao-Hua Zhang, Sheng-Zhong Duan, Feng Jia

**Affiliations:** ^1^Department of Neurosurgery, Ren Ji Hospital, Shanghai Jiao Tong University School of Medicine, Shanghai, China; ^2^Laboratory of Oral Microbiota and Systemic Diseases, Shanghai Ninth People’s Hospital, College of Stomatology, Shanghai Jiao Tong University School of Medicine, Shanghai, China; ^3^Shanghai Key Laboratory of Stomatology, National Center for Stomatology, National Clinical Research Center for Oral Diseases, Shanghai, China; ^4^Department of Periodontology, Shanghai Ninth People’s Hospital, Shanghai Jiao Tong University School of Medicine, Shanghai, China; ^5^Department of Neurosurgery, Nantong First People’s Hospital, The Second Affiliated Hospital of Nantong University, Nantong, China

**Keywords:** ischemic stroke, periodontitis, salivary microbiota, gut dysbiosis, IL-17A, neuroinflammation

## Abstract

Although epidemiological studies suggest that periodontitis is tightly associated with ischemic stroke, its impact on ischemic stroke and the underlysing mechanisms are poorly understood. Recent studies have shown that alteration in gut microbiota composition influences the outcomes of ischemic stroke. In the state of periodontitis, many oral pathogenic bacteria in the saliva are swallowed and transmitted to the gut. However, the role of periodontitis microbiota in the pathogenesis and progression of ischemic stroke is unclear. Therefore, we hypothesized that the periodontitis salivary microbiota influences the gut immune system and aggravates ischemic stroke. Mice receiving gavage of periodontitis salivary microbiota showed significantly worse stroke outcomes. And these mice also manifested more severe neuroinflammation, with higher infiltration of inflammatory cells and expression of inflammatory cytokines in the ischemic brain. More accumulation of Th17 cells and IL-17^+^ γδ T cells were observed in the ileum. And in Kaede transgenic mice after photoconversion. Migration of CD4^+^ T cells and γδ T cells from the ileum to the brain was observed after ischemic stroke in photoconverted Kaede transgenic mice. Furthermore, the worse stroke outcome was abolished in the IL-17A knockout mice. These findings suggest that periodontitis salivary microbiota increased IL-17A-producing immune cells in the gut, likely promoted the migration of these cells from the gut to the brain, and subsequently provoked neuroinflammation after ischemic stroke. These findings have revealed the role of periodontitis in ischemic stroke through the gut and provided new insights into the worse outcome of ischemic stroke coexisting with periodontitis in clinical trials.

## Introduction

Ischemic stroke is a worldwide cause of death and disability with limited treatment approaches (Campbell et al., 2019). In the past decades, a variety of studies have suggested that periodontitis is an independent risk factor for ischemic stroke (Wu et al., 2000; Elter et al., 2003; Jimenez et al., 2009). In addition, coexisting periodontitis in patients with ischemic stroke has been reported to have greater neurological deficit (Slowik et al., 2010). A previous study also reported that periodontitis increased neuroinflammation in mice after ischemic stroke (Chi et al., 2019). However, the mechanisms have remained incompletely understood.

Periodontitis is a periodontal disease that has an estimated prevalence of 20–50% in the general population (Albandar and Rams, 2002; Nazir, 2017). In the progression of periodontitis, dysbiosis of oral microbiota not only induces the loss of the gingiva, bone and ligament, but also significantly contribute to systemic inflammation (Kinane et al., 2017). Accumulating evidence has demonstrated that periodontitis is a risk factor for many systemic diseases, including cardio-cerebrovascular diseases (Leira et al., 2017; Czesnikiewicz-Guzik et al., 2019), metabolic diseases (Preshaw et al., 2012), and autoimmune diseases (Bunte and Beikler, 2019; De Luca and Shoenfeld, 2019).

Interorgan communications such as the “oral-gut” axis and “gut-brain” axis have gained increasing attention in recent years. In the “oral-gut” axis, the oral cavity is anatomically connected with the gut. It has been reported that approximately 150 genera and 700 species of microbes are colonized in the oral cavity (Gao et al., 2018). An adult human being produces 1–1.5 L of saliva every day, and about 10^11^ oral bacteria are flushed into the intestine with saliva (Richardson and Jones, 1958; Rashidi et al., 2021). One study has shown that 89% of the duodenal bacteria exist in matched oral samples, suggesting that oral bacteria can directly transmit to the duodenum (Barlow et al., 2021). In the state of periodontitis, many pathogenic bacteria, such as *Porphyromonas gingivalis*, *Tannerella forsythia*, *Fusobacterium nucleatum*, *Filifactor alocis*, *Streptococcus mitis/parasanguinis*, and *Parvimonas micra*, can enter the intestine with saliva (Lundmark et al., 2019; Belstrøm, 2020). These periodontitis salivary microbiotas may cause the dysbiosis of microbiota in the gut and contribute to the progression of many system diseases (Kitamoto et al., 2020; Ray, 2020). For example, periodontitis causes expansion of oral pathogenic bacteria that in turn aggravates colitis through intestinal colonization and migration of Th17 cells (Kitamoto et al., 2020). Oral pathogens may exacerbate liver disease through induction of gut dysbiosis and impairment of gut permeability (Acharya et al., 2017). In addition, periodontal pathogens, directly or indirectly, increase the citrullination burden and cause gut dysbiosis, leading to the increase of Th1, Th17 cells and pro-inflammatory cytokines in the gut, and eventually contributing to the aggravation of rheumatoid arthritis (du Teil Espina et al., 2019; Möller et al., 2020).

The importance of the “gut-brain” axis in neurologic diseases is reflected by the extensive communications between gut microbes and the central nervous system through the immune, endocrine, systemic, and neuronal pathways (Agirman and Hsiao, 2021). It has been shown that gut dysbiosis affects many brain disorders, including autism, anxiety, schizophrenia, multiple sclerosis, Parkinson’s disease, Alzheimer’s disease, and ischemic stroke (Cryan et al., 2019; Durgan et al., 2019). Antibiotic treatment changes the commensal gut bacteria and induces a neuroprotective effect in mice after stroke (Benakis et al., 2016, 2020). Transplant with fecal microbiota of young mice improves the recovery of stroke in old mice (Spychala et al., 2018). Furthermore, rapid dysbiosis of the gut microbiota induced by ischemic stroke exacerbates brain infarction in turn (Xu et al., 2021).

As described above, oral pathogens may have a profound impact on the permeability, homeostasis and immune response of the gut, which in turn has been demonstrated to play a critical role in ischemic stroke. Therefore, we speculate that periodontitis may affect ischemic stroke through dysbiosis of microbiota in the gut. We aimed to explore the potential “oral microbiota-gut-brain” axis in ischemic stroke in this study. We first established a mouse model that combined gavage of periodontitis salivary microbiota and ischemic stroke in mice. Then, we analyzed the outcome of stroke and change of immune cells in the brain and gut, and revealed significant alterations of IL-17A-producing cells. Finally, we tested the importance of IL-17A-producing cells in the association between periodontitis salivary microbiota and ischemic stroke using IL-17A knockout mice.

## Materials and Methods

### Collection of Saliva Sample

The protocol was approved by the Institutional Review and Ethics Board of Shanghai Ninth People’s Hospital, Shanghai Jiao Tong University School of Medicine. All medical data and samples were collected according to standard clinical procedures. Periodontitis were initially diagnosed by dentist (L Bai) and the patients we selected also met the following criteria (Kinane et al., 2017; Qian et al., 2021): (1) gum bleeding within 15 s after probing; (2) at least one site probing depth > 6 mm; (3) at least one site with attachment loss > 5 mm; (4) at least one site with alveolar bone absorption more than 1/2 of the length of the root. Patients who had taken any antibiotic or probiotic, smoked, undergone periodontal therapy in the previous 6 months, or had severe systemic diseases were not included. At the same time, healthy people were also recruited. All participants fasted for 2 h before the examination. Saliva samples of healthy people and periodontitis patients were collected and then mixed, respectively, and stored at −80°C until further processing.

### Animals

All animal experiments were approved by the Institutional Review and Ethics Board of Shanghai Ninth People’s Hospital, Shanghai Jiao Tong University School of Medicine. C57BL/6 mice were purchased from Charles River Laboratory Co., Ltd. (Beijing, China) and acclimatized the mice to the animal facility for 1 week before being randomly assigned to the experimental group. Il17a^–/–^ mice on the C57BL/6 background were kindly provided by You-Cun Qian (Chinese Academy of Sciences, Shanghai). Kaede transgenic mice were a generous gift from M. Tomura (Kyoto University). All mice were males (age = 6–8 weeks). Animals were kept under standard specific pathogen free (SPF) conditions at an appropriate temperature (22 ± 2°C) and humidity of 60% under a 12 h light/dark cycle. Food and water were available *ad libitum*. Genetically modified lines were bred in our facility. To minimize the impact of the maternal body on the composition of the microbiota, mice from different litters were mixed and then randomly assigned to the experimental group.

### Antibiotic Treatment

Ampicillin (Aladdin, Shanghai, China), metronidazole (Sigma-Aldrich, MO, United States), neomycin sulfate (Sigma-Aldrich), and vancomycin (Sigma-Aldrich), abbreviated AMNV. AMNV was administrated by drinking for 2 weeks, and 200 μl of the AMNV was administered by oral gavage every day during the second week. Concentration of drinking: ampicillin (0.3 g/L), metronidazole (0.3 g/L), neomycin sulfate (0.3 g/L), and vancomycin (0.15 g/L). Concentration of gavage: ampicillin (1 g/L), metronidazole (1 g/L), neomycin sulfate (1 g/L), and vancomycin (0.5 g/L).

### Middle Cerebral Artery Occlusion

The model was induced as previously described (Belayev et al., 1996). Briefly, the mice were under intraperitoneal anesthesia with 2% avertin (Sigma-Aldrich). The temperature was maintained using a homoeothermic blanket. The neck skin was cut along the midline. After the left common carotid artery, the internal carotid artery, and the external carotid artery separated carefully, a silicone-coated suture (Yushun, Henan, China) was inserted into the left external carotid artery, advanced into the internal carotid artery and wedged into the cerebral arterial circle to obstruct the origin of the middle cerebral artery. After 1 h of occlusion, the suture was withdrawn. Mice in the SHAM group underwent the same surgical procedures, but the suture was withdrawn immediately after the suture reached the origin of the middle cerebral artery.

### Measurement of Infarct Volume

Mice were euthanized 1 day after MCAO. Brains were immediately removed and frozen at −20°C for 30 min. Then mouse brains were coronally cut into 2 mm-thick sections. The sections were incubated with 2% 2, 3, 5-triphenyl-2H-tetrazolium chloride (TTC) (Sigma-Aldrich) at 37°C for 15 min and then photographed. Infarct size and volume were calculated by ImageJ software (National Institutes of Health, United States). To exclude the effects of brain edema, the relative percentage of infarct volume in hemisphere was calculated as follows: (volume of the contralateral hemisphere minus the volume of the non-lesioned ipsilateral hemisphere)/(volume of the contralateral hemisphere) (Lu et al., 2013).

### Neurological Deficit Scores

The Modified Garcia Score was used to measure motor function: body proprioception, forelimb walking, climbing, lateral turning, and limb symmetry as previously described (Wang et al., 2018). Each test was scored from 0 to 3 (maximal score 15).

### Quantitative Real-Time PCR (qPCR)

Mice were euthanized 1 day after MCAO, the ipsilateral hemisphere samples were collected. Total RNA was isolated from the ipsilateral hemisphere using Trizol (Thermo Fisher Scientific, MA, United States). cDNA was synthesized from RNA by using reverse transcription kits (Takara, Tokyo, Japan) and then detected by SYBR Green Mix (Thermo Fisher Scientific, Carlsbad, CA, United States) on a LightCycler 480 II (Roche, Switzerland). Gene expression was normalized by GAPDH. Primer sequences are listed in Table 1.

**TABLE 1:** Primer sequences for qPCR.

Gene	Forward primer (5′–3′)	Reverse primer (5′–3′)
*Il-1*β	AAGAGCTTCAGGCAGGCAGTATCA	TGCAGCTGTCTAATGGGAACGTA
*Tnf*-α	GCACAGAAAGCATGACCCG	GCCCCCCATCTTTTGGG
*Cxcl1*	CTGGGATTCACCTCAAGAACATC	CAGGGTCAAGGCAAGCCTC
*Cxcl2*	CCAACCACCAGGCTACAGG	GCGTCACACTCAAGCTCTG
*Ccl2*	TTAAAAACCTGGATCGGAACCAA	GCATTAGCTTCAGATTTACGGGT
*GAPDH*	ACCCAGAAGACTGTGGATGG	CACATTGGGGGTAGGAACAC

### Quantification of 16S rRNA Copies

Stool sample were collected and stored at −80°C. Frozen stools were weighed and extracted DNA through the Bacterial DNA Kit (Tiangen Biotech, Beijing, China). qPCR was used for quantification of 16S ribosomal (r16S) DNA copy numbers as previously described (Jimeno et al., 2018). The concentration of DNA samples was adjusted to equivalent. r16S DNA sequences were amplified from stool DNA using 0.2 μmol/L of the universal bacterial r16S gene primers Eubacteria-F primer (5′ ACTCCTACGGGAGGCAGCAGT 3′) and Eubacteria-R primer (5′ ATTACCGCGGCTGCTGGC 3′) in conjunction with the SYBR Green mix. The standard curve was prepared using a plasmid containing a V3-V4 DNA fragment.

The frozen saliva is dissolved and passed through a 0.22 μm filter to obtain filtrate. DNA of saliva and its filtrate was extracted through the Bacterial DNA Kit. The way to detected r16S DNA copy numbers was described above.

Specific periodontal bacteria were also detected by qPCR in mouse stools. DNA of stools was extracted through the Bacterial DNA Kit and the concentration of DNA was measured through NanoDrop Lite UV-Vis spectrophotometer (Thermo Scientific, United States). Then, DNA samples were adjusted to the same concentration. Sequences were amplified from 1 μl stool DNA sample using 0.2 μmol/L of the specific bacterial primers (Supplementary Table 1) in conjunction with the SYBR Green mix on a LightCycler 480 II.

### Enzyme Linked Immunosorbent Assay

The level of inflammatory cytokines (IL-1β and TNF-α) were detected by enzyme linked immunosorbent assay (ELISA) kits (JL10484 and JL18442, Jiang Lai Biotechnology, Shanghai, China) following the manufacturer’s instructions.

### Flow Cytometry

For surface marker analysis of the immune cells of ileum and the colon, cell suspensions were incubated with anti-CD16/CD32 antibody (101320, Biolegend, San Diego, CA, United States) for 10 min at 4°C to block the non-specific binding. Then cells were stained with the appropriate antibodies for 25 min at 4°C.

For surface marker analysis of the immune cells of brain, samples were subjected to Percoll (17089109, Civata, Uppsala, Sweden) gradient centrifugation before flow cytometric staining. Cell suspensions were incubated with anti-CD16/CD32 antibody for 10 min at 4°C to block the non-specific binding. Then cells were stained with the appropriate antibodies for 25 min at 4°C.

The following antibodies were used for staining: CD45 (553079, BD Biosciences, San Jose, CA, United States; 103115, Biolegend), CD4 (100406, 100407, Biolegend), CD8 (100734, Biolegend), TCR-γδ (118108, 128123, Biolegend), B220 (103212, Biolegend), CD11b (101228, Biolegend), CD64 (141712, Biolegend), MHCII (107607, Biolegend), CD11c (117306, Biolegend), Ly6G (127607, Biolegend), CD86 (560582, BD), CD206 (141712, Biolegend).

For intracellular staining of nuclear hormone receptor retinoid-related orphan receptor γt (RORγt) and forkhead box protein P3 (Foxp3) in the ileum and the colon, cells were first stained for surface markers as detailed above. Then, the FIX and PERM cell fixation kit (00-5123-43, Invitrogen, CA, United States) was incubated with the cells for 40 min at 4°C. Next, permeabilisation kit (00-833-56, Invitrogen) was used with the antibody RORγt (562607, BD) and the antibody Foxp3 (17-5773-80, eBioscience, San Diego, CA, United States) for 30 min at 4°C during the cells staining.

For intracellular cytokine staining, cells were first placed in culture with cell activation cocktail (with Brefeldin A) (423303, biolegend) for 5 h. Cells were stained for surface markers as detailed above. Then, the FIX and PERM cell fixation kit (00-5123-43, Invitrogen, CA, United States) was incubated with the cells for 40 min at 4°C. Next, permeabilisation kit (Invitrogen) was used with the antibody IL-17A (506916, Biolegend) for 30 min at 4°C during the cells staining.

Zombie (423107, Biolegend) was labeled to identify dead and live cells. Appropriate isotype-matched controls from the same vendors were included in intracellular staining in order to ensure proper compensation and staining specificity. Analysis was performed with FlowJo software (version 10, Tree Star).

### Immunofluorescence

Mice were euthanized 1 day after MCAO. Brains were removed following perfusion with saline and 4% paraformaldehyde (Biosharp, Guangzhou, China) in phosphate buffered saline (PBS) and then soaked in 30% sucrose in PBS. The brains were embedded in OCT (Sakura Finetek, United States) solution after sinking to the bottom of the liquid, and cryosections were prepared. Then brains were cut on a freezing microtome into 25 μm-thick sections and subjected to immunofluorescence staining. After antigen retrieval treatment, sections were incubated with a blocking buffer containing 5% normal goat serum and 0.3% Triton X-100 (Thermo Fisher Scientific) at 37°C for 1 h. Then the sections were incubated with anti-Iba1 (ab178846, Abcam, United States) or GFAP (16825-1-AP, Proteintech, United States) at 4°C overnight, followed by Fluorochrome-conjugated secondary antibodies (Thermo Fisher Scientific) at room temperature for 2 h the next day. Finally, the sections were counterstained with DAPI (Thermo Fisher Scientific). Images were captured using a fluorescence microscope (Leica, Germany).

### Photoconversion

The mice were anesthetized with 2% isoflurane (vol/vol), delivered 30% O_2_ and 70% N_2_O at a rate of 2 L/min, and kept at 37°C during the entire process. Photoconversion was performed by using a defocused violet laser source (405 nm, ZhongShanZiGu, China). The mice were placed in the supine position with a 2 cm incision in the abdomen. After exposing the distal small intestine, the body and the rest of the intestines were covered with aluminum foil. Intestinal tissues were applied with saline to maintain moisture. The exposed intestine was irradiated for 10 min and then returned to the abdominal cavity, and the peritoneum and skin were sutured.

### High-Throughput Sequencing and Processing

Extraction of DNA used the OMEGA Soil DNA Kit (M5635-02, Omega Bio-Tek, Norcross, GA, United States) following the manufacturer’s instructions. NanoDrop NC2000 spectrophotometer (Thermo Fisher Scientific, Waltham, MA, United States) and agarose gel electrophoresis were used to test the quantity and quality of extracted DNA, respectively.

PCR amplification of the near full-length bacterial 16S rRNA genes was performed using the forward primer 27F (5′-AGAGTTTGATCMTGGCTCAG-3′) and the reverse primer 1492R (5′-ACCTTGTTACGACTT-3′). The PCR products were quantified with PicoGreen dsDNA Assay Kit (Invitrogen, Carlsbad, United States) and sequenced on PacBio Sequel platform at Shanghai Personal Biotechnology Co., Ltd. (Shanghai, China). Microbiome bioinformatics were performed on QIIME2 platform with slight modification (Bolyen et al., 2019). Analysis of sequencing data was based on amplicon sequence variants (ASVs) (Bokulich et al., 2018). After chimera detection, high-quality sequences with 97% similarity were clustered into the same ASV. Classification of ASVs was performed based on the Greengenes Database.

Simpson’s diversity index was calculated using the ASV table in QIIME2, and visualized as box plots. Beta diversity was analyzed using Bray-Curtis metrics and visualized *via* non-metric multidimensional scaling. Heat map were clustered by UPGMA (default clustering algorithm) (Ramette, 2007) according to the Euclidean distance of the species composition data, and are arranged according to the clustering results by default; otherwise, they are arranged according to the default order of samples/groups.

### Statistics

Quantifications of infarct volume, bacteria content, Iba-1 or GFAP-positive cells in immunofluorescence staining, mRNA expression, protein expression, and immune cells in flow cytometry were represented in graphics with the mean value ± *SD*. Statistical analysis was performed using Prism (GraphPad Software, La Jolla, CA, United States). The differences between means of two experimental groups were analyzed by unpaired Student’s *t*-test or non-parametric test. The differences between means of three or more experimental groups were analyzed by One-Way ANOVA (Analysis of variance). Values of *p* ≤ 0.05 were considered statistically significant.

## Results

### Gavage of Periodontitis Salivary Microbiota Aggravates Ischemic Stroke in Mice

**FIGURE 1:**
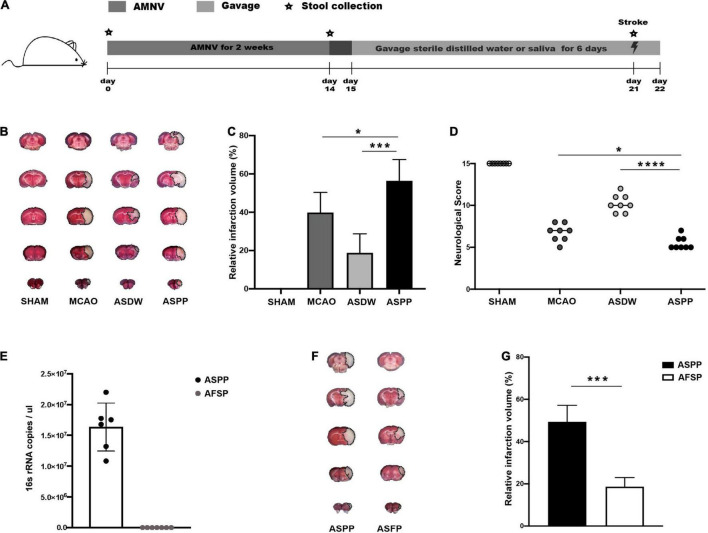
Gavage of periodontitis salivary microbiota aggravates ischemic stroke in mice. **(A)** Experimental design for testing the effects of saliva of periodontitis patients. AMNV, ampicillin, metronidazole, neomycin sulfate, and vancomycin. **(B)** Representative TTC-stained images of mouse cerebral sections 1 day after stroke. TTC, 2, 3, 5-triphenyl-2H-tetrazolium chloride. MCAO, middle cerebral artery occlusion. ASDW, antibiotics + sterile distilled water + MCAO. ASPP, antibiotics + saliva of periodontitis patients + MCAO. **(C)** Quantification of infarct volume. *n* = 8:8. **(D)** Neurological scores of mice 1 day after stroke. *n* = 8:8:8:8. **(E)** qPCR analysis of 16s rRNA gene copies in 1ul saliva and its filtrate. AFSP, antibiotics + filtrate of the saliva of periodontitis patients + MCAO. *n* = 6:7. **(F)** Representative TTC-stained images of mouse cerebral sections 1 day after stroke. **(G)** Quantification of infarct volume. *n* = 5:5. Values represent mean ± *SD*. One-Way ANOVA was used for statistical analysis in **(C,D)**, and Student’s *t*-test was used in **(G)**. *p < 0.05, ***p < 0.001, ****p < 0.0001.

We designed a protocol to explore the effects of periodontitis salivary microbiota in ischemic stroke (Figure 1A). We used a cocktail of antibiotics AMNV to deplete the endogenous microbiota of mice (Figure 1A). The mice were then treated with sterile distilled water (ASDW) or saliva of periodontitis patients (ASPP) by gavage before being subjected to MCAO (Figure 1B). Assessment by TTC staining 1 day after MCAO demonstrated that the ASPP group had significantly larger cerebral infarct volume than the MCAO and ASDW group (Figures 1B,C). Modified Garcia Score showed significantly lower neurological scores in the ASPP group compared to the MCAO and ASDW group (Figure 1D). These results indicated that the gavage of saliva of periodontitis patients aggravated ischemic stroke in mice. Body weights and the fecal bacterial density were both comparable between the ASDW and the ASPP group (Supplementary Figures 1A,B).

To find out whether microorganisms in the saliva of periodontitis patients caused worse ischemic stroke outcomes, we first got the filtrate of saliva through the bacterial filter and tested the density of bacterial content in the saliva and filtrate by qPCR, the result showed bacteria were not detectable in the filtrate (Figure 1E). Then, we established MCAO model after mice were treated with AMNV and gavage of filtrate of the saliva of periodontitis patients (AFSP). The AFSP group manifested markedly smaller infarct volume than the ASPP group, suggesting that salivary microbiota mediated the effects of saliva on ischemic stroke (Figures 1F,G). Furthermore, we found that gavage of salivary microbiota of healthy individuals did not affect ischemic stroke in mice (Supplementary Figures 1C,D). Similar to previously reported (Lundmark et al., 2019; Belstrøm, 2020), the periodontitis salivary microbiota presented a distinct microbiota profile from that of healthy individuals in our study, with enrichment of periodontal pathogenic bacteria such as *Prevotella intermedia, Prevotella oris, Fusobacterium nucleatum, Porphyromonas gingivalis, Porphyromonas endodontalis*, and *Tannerella forsythia* (Supplementary Figures 1E–G). To further explore the changes of periodontitis-associated microbiota in the gut after saliva gavage, we tested these bacteria in feces and found that *Prevotella intermedia*, *Fusobacterium nucleatum*, *Porphyromonas gingivalis*, and *Porphyromonas endodontalis* were increased in the feces (Supplementary Figure 1H).

### Gavage of Periodontitis Salivary Microbiota Promotes Activation of Microglia/Macrophages and Astrocytes in the Ischemic Region

**FIGURE 2:**
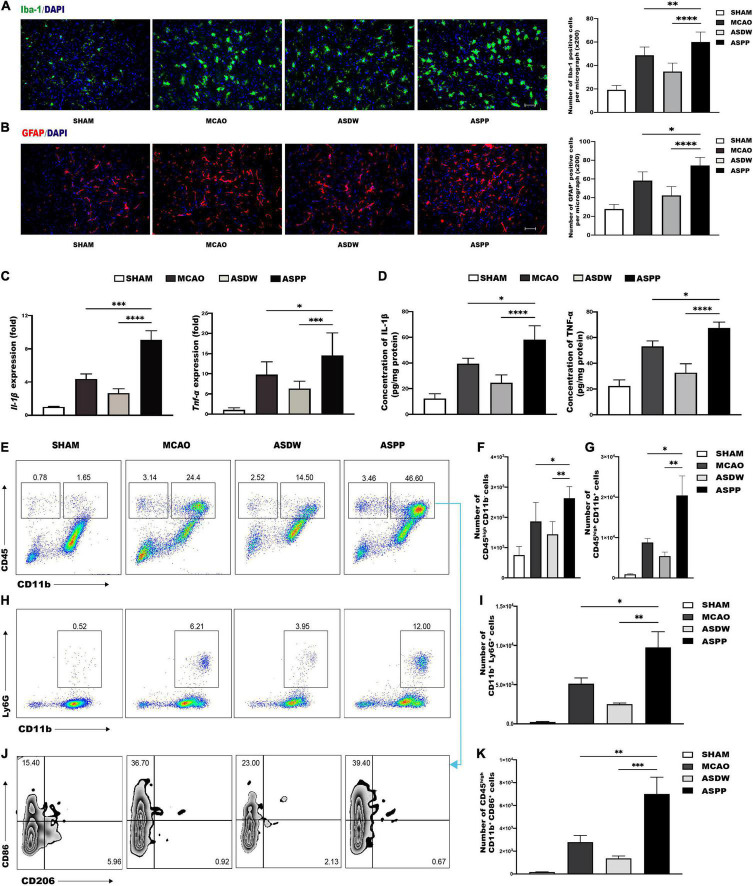
Gavage of periodontitis salivary microbiota promotes inflammation in the ischemic region. **(A)** Left, representative immunofluorescence staining of Iba-1 in the ischemic region of the brain. Scale bar = 100 μm. Right, quantification of Iba-1-positive cells. *n* = 5:5:5:5. **(B)** Left, representative immunofluorescence staining of GFAP in the ischemic region of the brain. Scale bar = 100 μm. Right, quantification of GFAP-positive cells. *n* = 5:5:5:5. **(C)** qPCR analysis of relative mRNA expression of Il-1β and Tnf-α in the ischemic brain. *n* = 7:12:8:10. **(D)** IL-1β and TNF-α detected by ELISA in the ischemic brain. *n* = 4:4:4:4. **(E)** Representative flow cytometry analysis of CD45^high^CD11b^–^ cells and CD45^high^CD11b^+^ cells in ischemic brain. **(F)** Quantification of CD45^high^CD11b^–^ cells. *n* = 6:6:6:6. **(G)** Quantification of CD45^high^CD11b^+^ cells. *n* = 6:6:6:6. **(H)** Representative flow cytometry analysis of CD11b^+^Ly6G^+^ cells. **(I)** Quantification of CD11b^+^Ly6G^+^ cells. *n* = 5:5:5:5. **(J)** Representative flow cytometry analysis of CD86 and CD206 in CD45^high^CD11b^+^ cells. **(K)** Quantification of CD45^high^CD11b^+^CD86^+^ cells. *n* = 6:6:6:6. Values represent mean ± SD. One-Way ANOVA was used for statistical analysis. *p < 0.05, ^**^p < 0.01, ^***^p < 0.001, ^****^p < 0.0001.

The inflammatory response following acute ischemic stroke is a critical element of brain damage. First, microglia/macrophage cells and astrocyte cells were analyzed by immunofluorescence staining of Iba-1and GFAP, respectively. The ASPP group had a significantly increased number of Iba-1^+^ cells and GFAP^+^ cells in the ischemic region compared to the MCAO and ASDW groups (Figures 2A,B). This result suggested that the more microglia/macrophages and astrocytes were rapidly activated in the ischemic region. Activated microglia/macrophage cells and astrocytes play a unique role in the secretion of pro-inflammatory cytokines. Results of qPCR and ELISA showed that the ASPP group had significantly increased expression of pro-inflammatory cytokines such as Interleukin-1β (Il-1β) and Tumor necrosis factor-alpha (Tnf-α) compared to the MCAO and ASDW groups (Figures 2C,D).

### Gavage of Periodontitis Salivary Microbiota Increases Infiltration of Immune Cells in the Ischemic Brain

Immune cells in the ischemic brain were further analyzed by using flow cytometry. Lymphocytes were identified as CD45^high^CD11b^–^ cells, macrophages or activated microglia were identified as CD45^high^CD11b^+^ cells, resting microglia were identified as CD45^int^CD11b^+^ cells, and neutrophils were identified as CD11b^+^Ly6G^+^ cells (Supplementary Figure 2A; Ponomarev et al., 2005). The ASPP group had a profound increase of lymphocytes (Figures 2E,F), macrophages/activated microglia (Figures 2E,G), and neutrophils (Figures 2H,I) in the ischemic brain compared to the MCAO and ASDW groups. The ASPP group has a decreased tendency in the number of resting microglia compared to the ASDW group (Supplementary Figure 3A).

We further analyzed the polarization of macrophages/microglia using CD86 as a marker for pro-inflammatory M1 and CD206 as a marker for anti-inflammatory M2 (Peng and Nixon, 2021). The ASPP group had a substantial increase in CD45^high^CD11b^+^CD86^+^ cells in the ischemic brain compared to the MCAO and ASDW group (Figures 2J,K). There was no significant difference in the number of CD45^int^CD11b^+^CD86^+^ cells between the ASPP group and the MCAO and ASDW group (Supplementary Figure 3B). CD206 was nearly not detectable in CD45^high^CD11b^+^ cells or CD45^int^CD11b^+^ cells after ischemic stroke (Figure 2J and Supplementary Figure 3B). These results indicated that gavage of periodontitis salivary microbiota induced more infiltration of immune cells in the ischemic brain, including lymphocytes, activated M1 population of microglia/macrophage, and neutrophils. In addition, we also tested these immune cells in ischemic brain of mice after gavage saliva of healthy people and did not detect significant increase of these immune cells (Supplementary Figures 3C,D).

### More Th17 Cells and IL-17^+^ γδ T Cells Accumulate to the Ischemic Brain After Gavage of Periodontitis Salivary Microbiota

**FIGURE 3:**
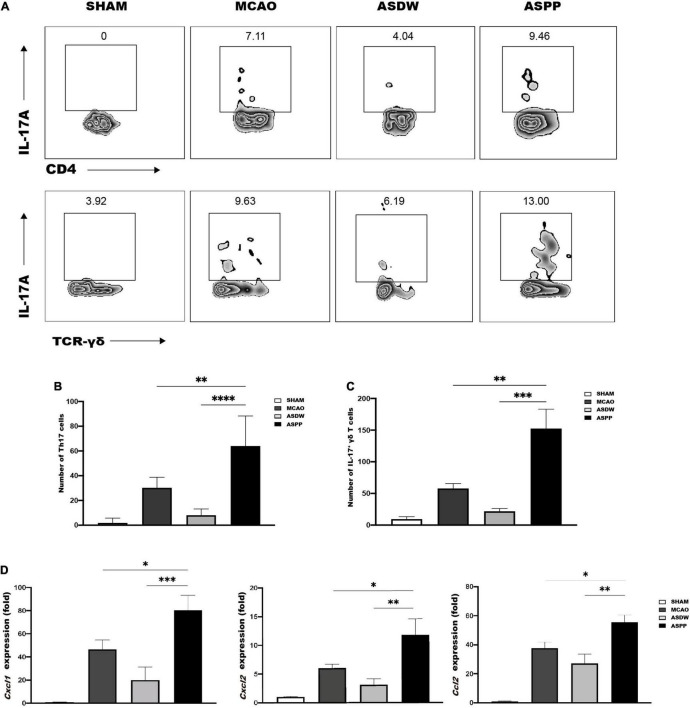
More Th17 and IL-17^+^ γδ T cells accumulate in the ischemic brain of ASPP group. **(A)** Representative flow cytometry analysis of Th17 cells and IL-17^+^ γδ T cells in the ischemic brain. **(B)** Quantification of Th17 cells. *n* = 4:4:4:4. **(C)** Quantification of IL-17^+^ γδ T cells. *n* = 5:5:5:5. **(D)** qPCR analysis of relative mRNA expression of *Cxcl1*, *Cxcl2*, and *Ccl2* in the ischemic brain. *n* = 7:10:8:10. Values represent mean ± *SD*. One-Way ANOVA was used for statistical analysis. *p < 0.05, ^**^p < 0.01, ^***^p < 0.001, ^****^p < 0.0001.

We next investigated the reason for the increased infiltration and the exacerbated inflammation in the ischemic brain induced by the gavage of periodontitis salivary microbiota. IL-17 is known to play a critical role in periodontitis and is tightly associated with dysbiosis of microbiota in the oral cavity (Cheng et al., 2014; Dutzan et al., 2018). Previous studies have indicated that oral pathogens may affect the expression of IL-17 in the gut (du Teil Espina et al., 2019; Feng et al., 2020; Kitamoto et al., 2020) and that IL-17-producing cells in the gut are closely associated with inflammatory response after ischemic stroke (Benakis et al., 2016). Therefore, we first used flow cytometry to detect IL-17-producing cells in the brain. Several studies have suggested that Th17 cells and IL-17^+^ γδ T cells were two major sources of IL-17A after ischemic stroke (Waisman et al., 2015; Zhang et al., 2021). In our experiment, Th17 cells were identified as CD4^+^IL17A^+^ cells and IL-17^+^ γδ T cells were identified as TCR-γδ^+^IL17A^+^ cells (Supplementary Figure 2B). Flow cytometry analysis revealed a significant increase of Th17 cells and IL-17^+^ γδ T cells in the ischemic brain of the ASPP group compared to the MCAO and ASDW group (Figures 3A–C). And we found gavage saliva of healthy people did not increase Th17 cells and IL-17^+^ γδ T cells in the ischemic brain compared to the ASDW group (Supplementary Figures 3E,F).

IL-17 has been shown to promote inflammatory cascade by inducing inflammatory chemokines to recruit other immune cells after stroke (Gelderblom et al., 2012; Waisman et al., 2015; Wojkowska et al., 2017; Zhang et al., 2021). Compared to the MCAO and ASDW groups, results of qPCR demonstrated that the ASPP group had significantly increased mRNA expression of chemokines, which was related to IL-17A, including C-X-C motif ligand 1 (*Cxcl1*), C-X-C motif ligand 2 (*Cxcl2*), and C-C chemokine ligand 2 (*Ccl2*) (Figure 3D). These results together indicated that gavage of periodontitis salivary microbiota elevated IL17-producing cells, which further induced chemokine production and the infiltration of other pro-inflammatory cells after ischemic stroke.

### Gavage of Periodontitis Salivary Microbiota Increases Th17 Cells and IL-17^+^ γδ T Cells in the Small Intestine

**FIGURE 4:**
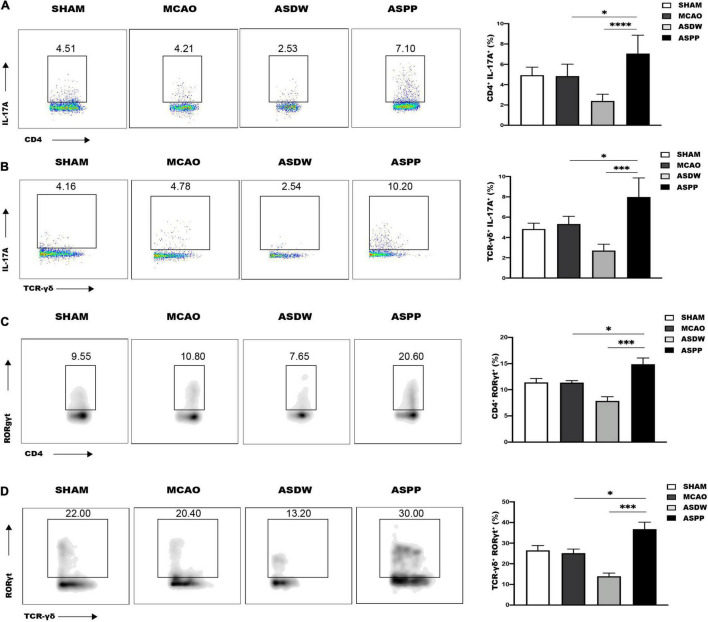
Gavage of periodontitis salivary microbiota increases Th17 and IL-17^+^ γδ T cells in the small intestine. **(A)** Left, representative flow cytometry analysis of CD4^+^IL-17A^+^ cells (Th17 cells) in the small intestine. Right, quantification of Th17 cells. *n* = 5:5:6:6. **(B)** Left, representative flow cytometry analysis of TCR-γδ^+^IL-17A^+^ cells (IL-17^+^ γδ T cells) in the small intestine. Right, quantification of IL-17^+^ γδ T cells. *n* = 5:5:5:5. **(C)** Left, representative flow cytometry analysis of CD4^+^RORγt^+^ T cells in the small intestine. Right, quantification of CD4^+^RORγt^+^ cells. *n* = 6:6:6:6. **(D)** Left, representative flow cytometry analysis of TCR-γδ^+^RORγt^+^ cells in the small intestine. Right, quantification TCR-γδ^+^RORγt^+^ cells. *n* = 5:5:5:5. Values represent mean ± *SD*. One-Way ANOVA was used for statistical analysis. *p < 0.05, ^***^p < 0.001, ^****^p < 0.0001.

We further hypothesized that the increased accumulation of IL17-producing cells in the ischemic brain originated from the gut after gavage of periodontitis salivary microbiota. Flow cytometry analysis showed the ASPP group had comparable CD4^+^ T cells and TCR-γδ^+^ T cells with the ASDW and MCAO groups in the small intestine and colon (Supplementary Figure 4). Meanwhile, flow cytometry analysis revealed a marked increase of Th17 cells and IL-17^+^ γδ T cells in the small intestine of ASPP group (Figures 4A,B), but not the colon (Supplementary Figures 5A,B). Next, we also found that gavage of periodontitis salivary microbiota also increased Th17 cells and IL-17^+^ γδ T cells in the small intestine of mice without ischemic stroke (Supplementary Figures 6A,B).

RORγt has been demonstrated to play an important role in intestinal homeostasis and promote the expression of IL-17 (Ivanov et al., 2006; Eberl, 2012; Kumar et al., 2021). Consistently, there was an increased expression of RORγt in CD4^+^ T cells and TCR-γδ^+^ T cells in the small intestine of ASPP group (Figures 4C,D), but not the colon (Supplementary Figures 5C,D). Treg cells express Foxp3 and are related to the alteration of IL-17 (Huber et al., 2011). However, CD4^+^Foxp3^+^ Treg cells were not significantly different in the small intestine among the groups (Supplementary Figure 6C,D). These results demonstrated that gavage of periodontitis salivary microbiota promoted IL-17-producing cells in the small intestine, which was similar to the changes in the ischemic brain, and the mechanism may be up-regulated expression of RORγt.

### Intestinal CD4^+^ T Cells and γδ T Cells Migrate to the Brain After Ischemic Stroke

**FIGURE 5:**
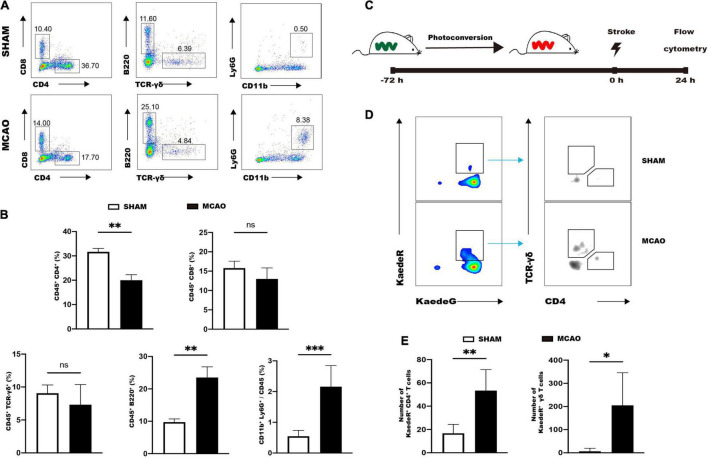
Intestinal immune cells rapidly respond to ischemic stroke. **(A)** Representative flow cytometry analysis of immune cells in the small intestine 1d after MCAO or sham operation. **(B)** Quantifications of immune cells in the small intestine. *n* = 5:5. **(C)** Strategy for analyzing intestinal immune cell trafficking to the brain by using Kaede transgenic mice. **(D)** Representative flow cytometry analysis of photoconverted CD4^+^ T cells and γδ T cells in the ischemic brain. **(E)** Quantifications of photoconverted CD4^+^ T cells and γδ T cells. *n* = 4:6. Values represent mean ± *SD*. Student’s *t*-test was used for statistical analysis. ns, not significant; *p < 0.05 ^**^p < 0.01, ^***^p < 0.001.

We then investigated the possibility of migration of immune cells from the gut to the brain in response to ischemic stroke. The MCAO group showed a significant decrease of CD4^+^ T cells in the small intestine 1 day after ischemic stroke compared to the SHAM group (Figures 5A,B). Conversely, the MCAO group manifested increases of CD11b^+^Ly6G^+^ neutrophils and CD45^+^B220^+^ B cells in the intestine compared to the SHAM group (Figures 5A,B). The other populations such as γδ T cells, CD8^+^ T cells, dendritic cells, and macrophage cells were comparable between MCAO and SHAM groups (Figures 5A,B and Supplementary Figures 7A,B). These results indicated that the reduced intestinal CD4^+^ T cells may migrate to the ischemic brain.

Kaede transgenic mice were utilized to further illustrate the migration of immune cells. Kaede transgenic mice express the Kaede fluorescent protein, which could achieve photoconversion from a green (KaedeG) to a red (KaedeR) fluorescence after being exposed to violet light (Tomura et al., 2008). Photoconversion was achieved by exposure of distal small intestines of Kaede transgenic mice to violet light (Figure 5C), and 72 h after photoconversion the mice were subjected to MCAO and sham operation. Immune cells of brain were analyzed by flow cytometry 24 h after stroke (Figure 5C). Flow cytometry analysis showed that MCAO significantly increased the numbers of KaedeR^+^CD4^+^ cells and KaedeR^+^TCR-γδ^+^ cells in the brain (Figures 5D,E).

These results suggested that intestinal immune cells rapidly responded to ischemic stroke and CD4^+^ T cells and γδ T cells could migrate to the brain after ischemic stroke. Meanwhile, we also tested the migration of macrophages. KaedeR^+^CD11b^+^CD64^+^ cells were not detectable in the brain of the SHAM and MCAO group, suggesting that macrophages did not migrate from the small intestine to the brain 24 h after ischemic stroke (Supplementary Figure 7C).

### IL-17A Is Indispensable for the Aggravation of Ischemic Stroke Induced by Gavage of Periodontitis Salivary Microbiota

**FIGURE 6:**
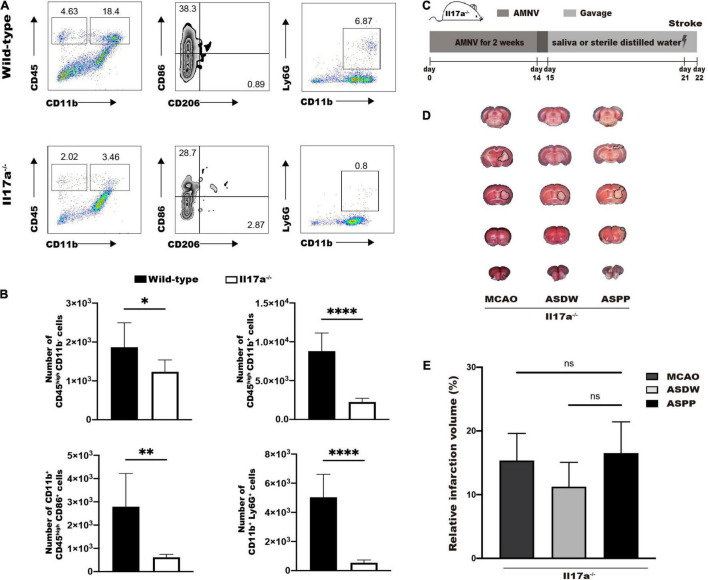
IL-17A is indispensable for the aggravation of ischemic stroke induced by gavage of periodontitis salivary microbiota. **(A)** Representative flow cytometry analysis of immune cells in ischemic brain of wild-type and Il17a^–/–^ mice after MCAO. **(B)** Quantifications of immune cells in **(A)**. *n* = 6:6:6:6. **(C)** Experimental design for testing the role of IL-17A in salivary microbiota-induced aggravation of ischemic stroke. **(D)** Representative TTC-stained image in Il17a^–/–^ mice 1 day after MCAO. **(E)** Quantification of infarct volume. *n* = 5:5:5. Values represent the mean ± *SD*. Student’s *t*-test was used for statistical analysis in **(B)**, and One-Way ANOVA was used in **(E)**. ns, not significant; *p < 0.05, ^**^p < 0.01, ^****^p < 0.0001.

Finally, we tested whether IL-17A was required for periodontitis salivary microbiota to exacerbate ischemic stroke. Wild-type mice and Il17a^–/–^ mice were subjected to MCAO and ischemic brains were analyzed by flow cytometry 24 h after stroke. Flow cytometry analysis showed significantly reduced number of CD45^high^CD11b^–^ cells, CD45^high^CD11b^+^ cells, CD45^high^CD11b^+^CD86^+^ cells, and CD11b^+^Ly6G^+^ cells in Il17a^–/–^ mice after MCAO (Figures 6A,B). These results indicated that IL-17A played a vital role in the mobilization of immune cells by MCAO to induce neuroinflammation.

Then we repeated the protocol on Il17a^–/–^ mice to explore the effects of periodontitis salivary microbiota in ischemic stroke (Figure 6C). TTC staining 1 day after MCAO demonstrated that the difference in cerebral infarct volume we previously observed in wildtype mice was disappeared in Il17a^–/–^ mice (Figures 6D,E). These results demonstrated IL-17A was required for the periodontitis salivary microbiota to exacerbate ischemic stroke.

## Discussion

**FIGURE 7:**
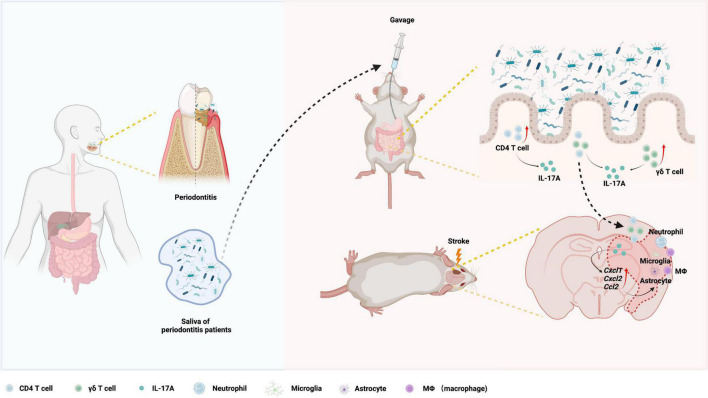
Working model for the exacerbation of ischemic stroke by periodontitis salivary microbiota. Periodontitis salivary microbiota increases IL-17A-producing cells in the small intestine, which contain CD4^+^ T cells and TCR-γδ^+^ T cells. Increased migration of IL-17A-producing cells from the gut to the brain elevates post-ischemic chemokines (*Cxcl1*, *Cxcl2*, and *Ccl2*) and ultimately infiltration and activation of immune cells (macrophages/microglia, astrocyte, and neutrophils) in ischemic brain, leading to exacerbated neuroinflammation and ischemic stroke phenotype.

Although periodontitis has been proven to be associated with ischemic stroke (Elter et al., 2003; Sen et al., 2018), the mechanisms remain incompletely understood. In this study, we revealed that gavage of periodontitis salivary microbiota aggravated ischemic stroke and neuroinflammation in mice. Periodontitis salivary microbiota increased IL-17A-producing immune cells in small intestine, and provoked migration of IL-17A-producing cells from the gut to the brain, which might initiate the early inflammatory cascade and ultimately exacerbated ischemic stroke. Finally, we demonstrated that IL-17A was required for the periodontitis salivary microbiota to exacerbate ischemic stroke (Figure 7).

We designed a protocol to explore the effects of periodontitis salivary microbiota. Cumulative clinical research data have implied a tight association between the oral microbiota and systemic diseases (Belstrøm, 2020). Most periodontitis models were induced by silk ligature or specific bacteria (Kesavalu et al., 2007; Marchesan et al., 2018), which induced chronic periodontal inflammation and systemic inflammation. However, because of the difference in oral microbiota between human beings and rodents, it is difficult to simulate the microbiota composition of human periodontitis-related oral pathogens in rodent models. A human oral microbiota-associated mouse model hinted a possibility that the salivary microbiota of humans could be transplanted into the gut of mice (Li et al., 2019). Furthermore, our data showed the periodontitis salivary microbiota contains a rich abundance of *Porphyromonas* and *Fusobacterium*, which could colonize and cause dysbiosis in the digestive tract (Li et al., 2019; Hong et al., 2021; Watanabe et al., 2021). Thus, the saliva of periodontitis patients was used to simulate the original microbiota in our experimental design.

Previous studies have demonstrated that disorders of the intestines can aggravate the inflammation of the central nervous system (Agirman et al., 2021). In our study, we discovered that the gavage of periodontitis salivary microbiota aggravated ischemic stroke and exacerbated inflammation in the ischemic brain. And excessively activated microglia/macrophages cells are related to neuronal damages. Microglia is an intrinsic immune cell of the central nervous system, its activation due to infiltration of peripheral immune cells revealed the cross-talk between the peripheral immune response in the immune activation of the central nervous system.

The mice treated with gavage of periodontitis salivary microbiota had more Th17 cells and IL-17^+^ γδT cells in the ischemic brain. It has been reported that these two groups of cells are the main source of IL-17A after ischemic stroke (Waisman et al., 2015; Zhang et al., 2021). IL-17A increases rapidly after ischemic stroke (Zhang et al., 2014) and plays a crucial role in aggravating cerebral infarction through various ways. First, IL-17A promotes neuronal apoptosis by upregulation of apoptotic proteins (Li et al., 2017). Second, IL-17A promotes the activation of microglia and astrocytes cells, as well as the production of inflammatory cytokines after ischemic stroke (Sonobe et al., 2008; Zhang et al., 2021). Third, IL-17A increases the expression of chemokines, such as *Cxcl1*, *Cxcl2*, *Cxcl9, Cxcl10*, *Ccl2*, *Ccl3*, and *Ccl20*, and further promotes the infiltration of immune cells (Waisman et al., 2015). Finally, IL-17A decreases the expression of tight junction proteins, further promoting infiltration of immune cells and impairment of the blood-brain barrier (Huppert et al., 2010; Ni et al., 2018). Our results showed that gavage of periodontitis saliva increased the expression of inflammatory factors, including *Il-1*β, *Tnf*-α, *Cxcl1, Cxcl2, and Ccl2*, in the ischemic brain in mice. Among these chemokines, *Cxcl1*is mainly induced by IL-17A and has a strong ability to recruit neutrophils (Gelderblom et al., 2012). Resultantly, our data also showed that gavage of periodontitis saliva significantly increased the accumulation of neutrophils, which have been considered as a pathologic hallmark of early ischemic stroke (Jickling et al., 2015; Perez-de-Puig et al., 2015).

It has been shown that the connections between periodontitis and several inflammatory diseases such as psoriasis, rheumatoid arthritis, and inflammatory bowel diseases are mediated by intestinal IL-17A (Bunte and Beikler, 2019). Our data showed that IL-17A also linked the microbiota of periodontitis and the worse outcome of ischemic stroke. IL-17A plays a vital role in the process of periodontitis because of its close association with periodontal bacteria (Dutzan et al., 2018). For example, a previous report has demonstrated that *Porphyromonas gingivalis* can increase the level of IL-17A in the oral cavity and intestinal tract (Sato et al., 2017). In our study, we found that gavage of periodontal pathogens, which included a rich abundance of *Prevotella intermedia*, *Prevotella oris*, *Fusobacterium nucleatum*, and *Porphyromonas gingivalis*, increased the number of IL-17A-producing cells in the small intestine. Furthermore, the small intestine and colon may have a different composition of bacteria (Li et al., 2020), which may have contributed to differential alterations in IL-17A between the small intestine and colon in our study. RORγt is related to microbiota and is considered as a key transcriptional regulator of IL-17A gene in T cells, including CD4^+^ T cells and γδT cells (Eberl, 2012; Kumar et al., 2021). In our study, the increased expression of RORγt may explain the reason of up-regulated IL-17A induced by periodontitis salivary microbiota.

Ischemic stroke could induce rapid gut response both in humans and mice (Yin et al., 2015; Xu et al., 2021). And ischemic stroke may lead to gut paralysis, barrier disruption, increased abundance of pathogens and decreased beneficial commensals, which may in turn exacerbate the ischemic stroke and form a vicious circle (Singh et al., 2016; Xu et al., 2021). In our study, a significant increased proportion of B cells and neutrophils in the small intestine after ischemic stroke demonstrated that the immune cells rapidly responded to ischemic stroke. Regulating the response of these immune cells may be a potential target for intervening in the vicious circle of intestinal flora disturbance and cerebral infarction injury. A previous study demonstrated that intestinal T cells traffic to the meninges after stroke in the early stage (Benakis et al., 2016), our experiments illustrated the migration of intestinal CD4^+^ T cells and γδ T cells to the region of ischemic brain. These rapidly immune responses in the gut further supported the importance of the gut-brain axis in the pathological process of ischemic stroke.

## Conclusion

In summary, periodontitis salivary microbiota exacerbates the outcome of ischemic stroke. This study provided mechanistic insights that the salivary microorganisms of periodontitis patients may exert their pathogenic effects in the gut to aggravate ischemic stroke through immunological mobilization. Besides, these findings have revealed the role of periodontitis in systemic disease and provide new insight into the worse outcome of stroke coexisting with periodontitis in clinical trials, and support that treatment of periodontitis is an important strategy to enhance prevention and therapeutic effectiveness of ischemic stroke.

## Data Availability Statement

The datasets presented in this study can be found in online repositories. The names of the repository/repositories and accession number(s) can be found below: https://www.ncbi.nlm.nih.gov/bioproject/PRJNA801456.

## Ethics Statement

The studies involving human participants were reviewed and approved by the Ethics Committee of Shanghai Ninth People’s Hospital, Shanghai Jiao Tong University School of Medicine. The patients/participants provided their written informed consent to participate in this study. The animal study was reviewed and approved by the Ethics Committee of Shanghai Ninth People’s Hospital, Shanghai Jiao Tong University School of Medicine.

## Author Contributions

Y-LC, LB, and DD conducted the research and wrote the manuscript. SS, CQ, TL, SX, and X-BB helped to complete the experiment. LJ-D, L-JZ, W-ZL, Y-CJ, and YL performed the statistical analysis and edited the manuscript. FJ, S-ZD, and X-HZ guided the entire study and provided the supervision and final check. All authors read the final version of the manuscript and approved it.

## Conflict of Interest

The authors declare that the research was conducted in the absence of any commercial or financial relationships that could be construed as a potential conflict of interest.

## Publisher’s Note

All claims expressed in this article are solely those of the authors and do not necessarily represent those of their affiliated organizations, or those of the publisher, the editors and the reviewers. Any product that may be evaluated in this article, or claim that may be made by its manufacturer, is not guaranteed or endorsed by the publisher.

## Supplementary Material

Supplementary Figure 1 **(A)** Body weight of mice during the experiment shown in Figure 1B. *n* = 8:8:8:8. **(B)** Measurements of bacterial density. **(C)** Representative TTC-stained images and **(D)** quantification of infarct volume in ASDW and ASHP groups 1 day after MCAO. *n* = 8:6. ASHP, after AMNV to deplete the endogenous microbiota of mice, the mice were then treated with saliva of healthy people by gavage before being subjected to MCAO. **(E)** Different salivary microbial composition between healthy individuals and periodontitis patients. α-diversity assessed by Simpson index. **(F)** β-diversity analysis through non-metric multidimensional scaling (NMDS) analysis. **(G)** Compositional heatmap of salivary microbiota detected by 16S rRNA gene sequencing. **(H)** Detection of periodontal bacteria in mouse feces by qPCR. Values represent mean ± *SD*. ns, not significant. **p* < 0.05, ^****^*p* < 0.0001.

Supplementary Figure 2 **(A)** Gating strategy for flow cytometry analysis of the brain immune cells. **(B)** Gating strategy for flow cytometry analysis of the intestinal immune cells.

Supplementary Figure 3 Flow cytometry analysis of brain immune cells. **(A)** Gavage of salivary microbiota of periodontitis patients does not significantly alter the number of CD45^int^CD11b^+^cells in the ischemic brain 1 day after ischemic stroke. Left, representative flow cytometry analysis of CD45^int^CD11b^+^ cells. Right, quantification of CD45^int^CD11b^+^ cells. *n* = 6:5:6:6. **(B)** Gavage of salivary microbiota of periodontitis patients does not significantly alter the number of CD45^int^CD11b^+^CD86^+^ cells in the ischemic brain 1 day after ischemic stroke. Left, representative flow cytometry analysis of CD45^int^CD11b^+^CD86^+^ cells. Right, quantification of CD45^int^CD11b^+^CD86^+^ cells. *n* = 6:5:6:6. **(C)** Representative flow cytometry analysis of immune cells in ischemic brain of the ASDW and ASHP group after MCAO. **(D)** Quantifications of immune cells in **(C)**. *n* = 6:6. **(E)** Representative flow cytometry analysis of Th17 cells and IL-17^+^ γδ T cells in the ischemic brain of the ASDW and ASHP group after MCAO. **(F)** Left, quantification of Th17 cells. *n* = 4:4 Right, quantification of IL-17^+^ γδ T cells. *n* = 5:5. Values represent mean ± *SD*. One-Way ANOVA was used for statistical analysis in **(A,B)**, and Student’s *t*-test was used in **(D,F)**. ns, not significant.

Supplementary Figure 4 Flow cytometry analysis of CD4^+^ T cells and TCR-γδ^+^ T cells. The ASPP group had comparable CD4^+^ T cells and TCR-γδ^+^ T cells with the ASDW and MCAO groups in the small intestine and colon. **(A)** Quantification of CD45^+^CD4^+^ cells in the small intestine. *n* = 5:5:6:6. **(B)** Quantification of CD45^+^TCR-γδ^+^ cells in the small intestine. *n* = 5:5:6:6. **(C)** Quantification of CD45^+^CD4^+^ cells in the colon. *n* = 5:5:5:5. **(D)** Quantification of CD45^+^TCR-γδ^+^ cells in the colon. *n* = 5:5:5:5. Values represent mean ± *SD*. One-Way ANOVA was used for statistical analysis. ns, not significant.

Supplementary Figure 5 Gavage of salivary microbiota of periodontitis patients does not affect Th17 cells or IL17^+^ γδ T cells in mouse colons after ischemic stroke. **(A)** Left, representative flow cytometry analysis of CD4^+^IL-17A^+^ cells (Th17 cells) in the colon. Right, quantification of Th17 cells. *n* = 5:5:6:6. **(B)** Left, representative flow cytometry analysis of TCR-γδ^+^IL-17A^+^ cells (IL-17^+^ γδ T cells) in the colon. Right, quantification of IL-17^+^γδ T cells. *n* = 5:5:5:5. **(C)** Left, representative flow cytometry analysis of CD4^+^RORγt^+^ T cells in the colon. Right, quantification of CD4^+^RORγt^+^ cells. *n* = 6:6:6:6. **(D)** Left, representative flow cytometry analysis of TCR-γδ^+^RORγt^+^ cells in the colon. Right, quantification TCR-γδ^+^RORγt^+^ cells. *n* = 5:5:5:5. Values represent mean ± *SD*. One-Way ANOVA was used for statistical analysis. ns, not significant.

Supplementary Figure 6 **(A)** Experimental design for testing the effects of saliva of periodontitis patients without MCAO. **(B)** Increasing of Th17 and IL-17^+^ γδ T cells in the small intestine didn’t require MCAO operation. Left, quantification of Th17 cells. *n* = 5: 6: 6 Right, quantification of IL-17^+^ γδ T cells. *n* = 5: 5: 5. **(C)** Gavage of salivary microbiota of periodontitis patients does not affect CD4^+^Foxp3^+^ cells in the small intestine of mice after ischemic stroke. Representative flow cytometry analysis of CD4^+^Foxp3^+^ cells. **(D)** Quantification of CD4^+^Foxp3^+^ cells in **(C)**. *n* = 6:6:6:6. Values represent mean ± *SD*. One-Way ANOVA was used for statistical analysis. ns, not significant. **p* < 0.05, ^**^*p* < 0.01, ^***^*p* < 0.001.

Supplementary Figure 7 Flow cytometry analysis of CD11c^+^MHCII^+^ cells and CD11b^+^CD64^+^ cells. MCAO does not affect dendritic cells or macrophage cells in the small intestine of mice after ischemic stroke. And macrophages do not migrate from the small intestine to the brain after ischemic stroke. **(A)** Representative flow cytometry analysis of CD11c^+^MHCII^+^ cells and CD11b^+^CD64^+^ cells in the small intestine. **(B)** Quantification of CD11c^+^MHCII^+^ cells and CD11b^+^CD64^+^ cells. *n* = 5: 4. **(C)** Representative flow cytometry analysis of KaedeR^+^CD11b^+^CD64^+^ macrophages in the brain of mice 1 day after MCAO or sham operation. Values represent mean ± *SD*. Student’s *t*-test was used for statistical analysis. ns, not significant.
