# Supplementary material for: Periodontitis Salivary Microbiota Aggravates Ischemic Stroke Through IL-17A
Source: Front Neurosci. 2022 May 19;16:876582. doi: 10.3389/fnins.2022.876582 (PMC9160974; doi:10.3389/fnins.2022.876582)
Supplement: Supplementary file 1 [file Table_1.docx]

**Supplementary Table 1：Specific bacteria primer sequences for qPCR**

| Bacteria | Forward primer (5′–3′) | Reverse primer (5′–3′) |
| --- | --- | --- |
| *P.intermedia* | CGGCCTAATACCCGATGTTG | CCCATCCTCCACCGATGA |
| *F. nucleatum* | AAGCGCGTCTAGGTGGTTATGT | TGTAGTTCCGCTTACCTCTCCAG |
| *P.gingivalis* | TAGCTTGCTAAGGTCGATGG | CAAGTGTATGCGGTTTTAGT |
| *P. endodontalis* | TATTGACAAGGCTGTGGCTACC | TTCTTCGTCCCCATTAGCCGA |
