# Supplementary figures and images for: Periodontitis Salivary Microbiota Aggravates Ischemic Stroke Through IL-17A
Source: Front Neurosci. 2022 May 19;16:876582. doi: 10.3389/fnins.2022.876582 (PMC9160974; doi:10.3389/fnins.2022.876582)

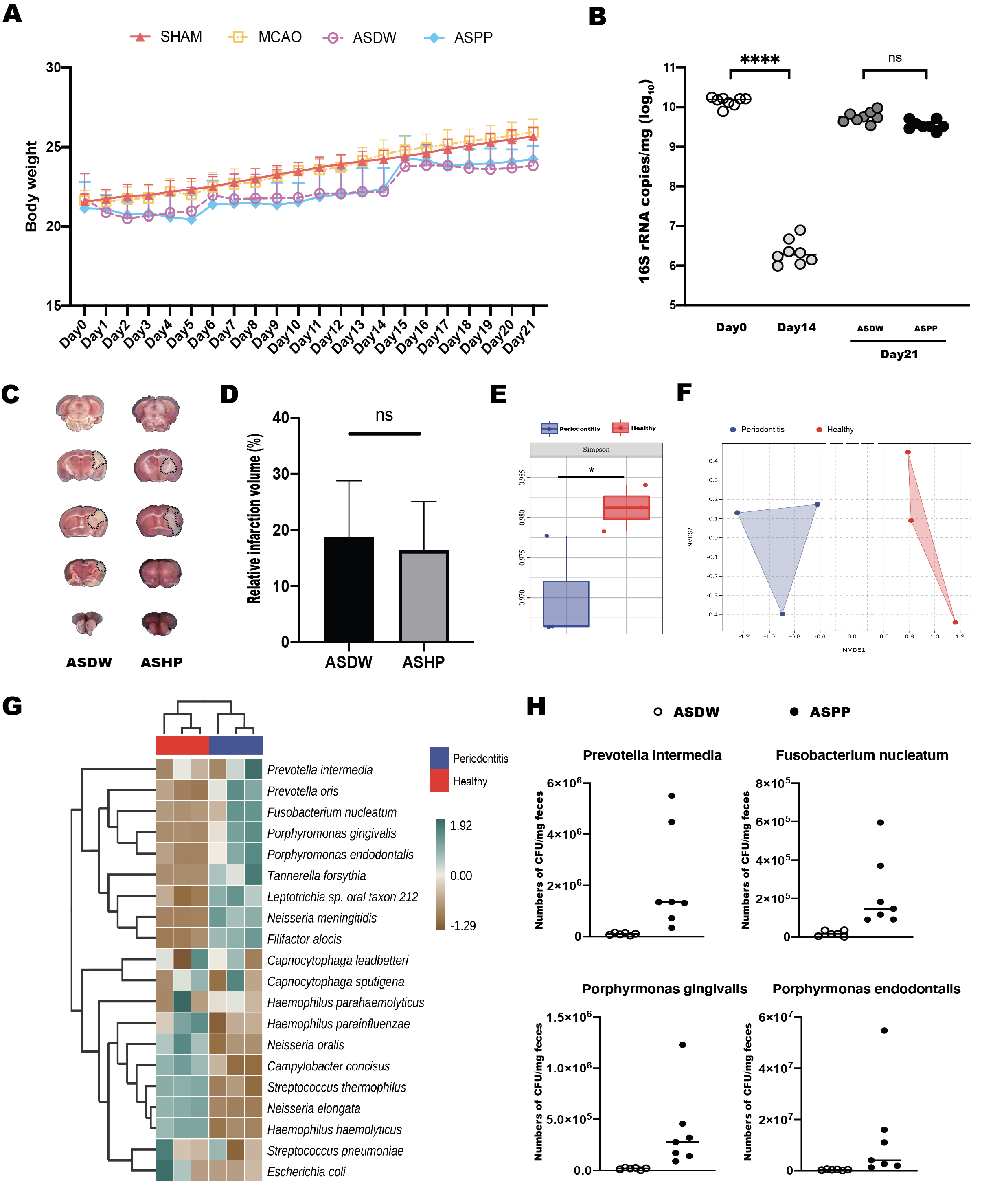

Supplement: Supplementary Figure 1 — (A) Body weight of mice during the experiment shown in Figure 1B. n = 8:8:8:8. (B) Measurements of bacterial density. (C) Representative TTC-stained images and (D) quantification of infarct volume in ASDW and ASHP groups 1 day after MCAO. n = 8:6. ASHP, after AMNV to deplete the endogenous microbiota of mice, the mice were then treated with saliva of healthy people by gavage before being subjected to MCAO. (E) Different salivary microbial composition between healthy individuals and periodontitis patients. α-diversity assessed by Simpson index. (F) β-diversity analysis through non-metric multidimensional scaling (NMDS) analysis. (G) Compositional heatmap of salivary microbiota detected by 16S rRNA gene sequencing. (H) Detection of periodontal bacteria in mouse feces by qPCR. Values represent mean ± SD. ns, not significant. *p < 0.05, ****p < 0.0001. [file Image_1.JPEG]

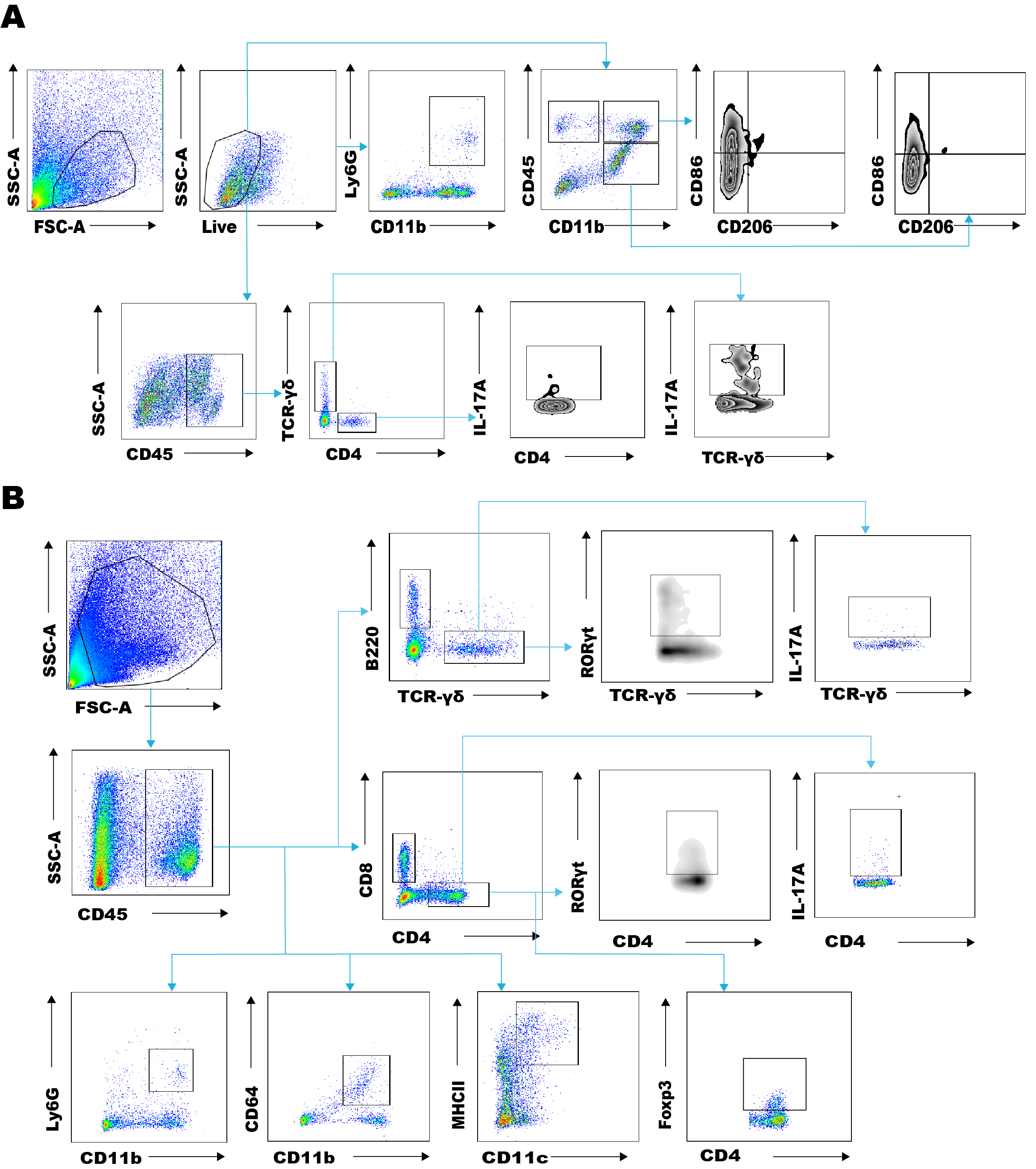

Supplement: Supplementary Figure 2 — (A) Gating strategy for flow cytometry analysis of the brain immune cells. (B) Gating strategy for flow cytometry analysis of the intestinal immune cells. [file Image_2.JPEG]

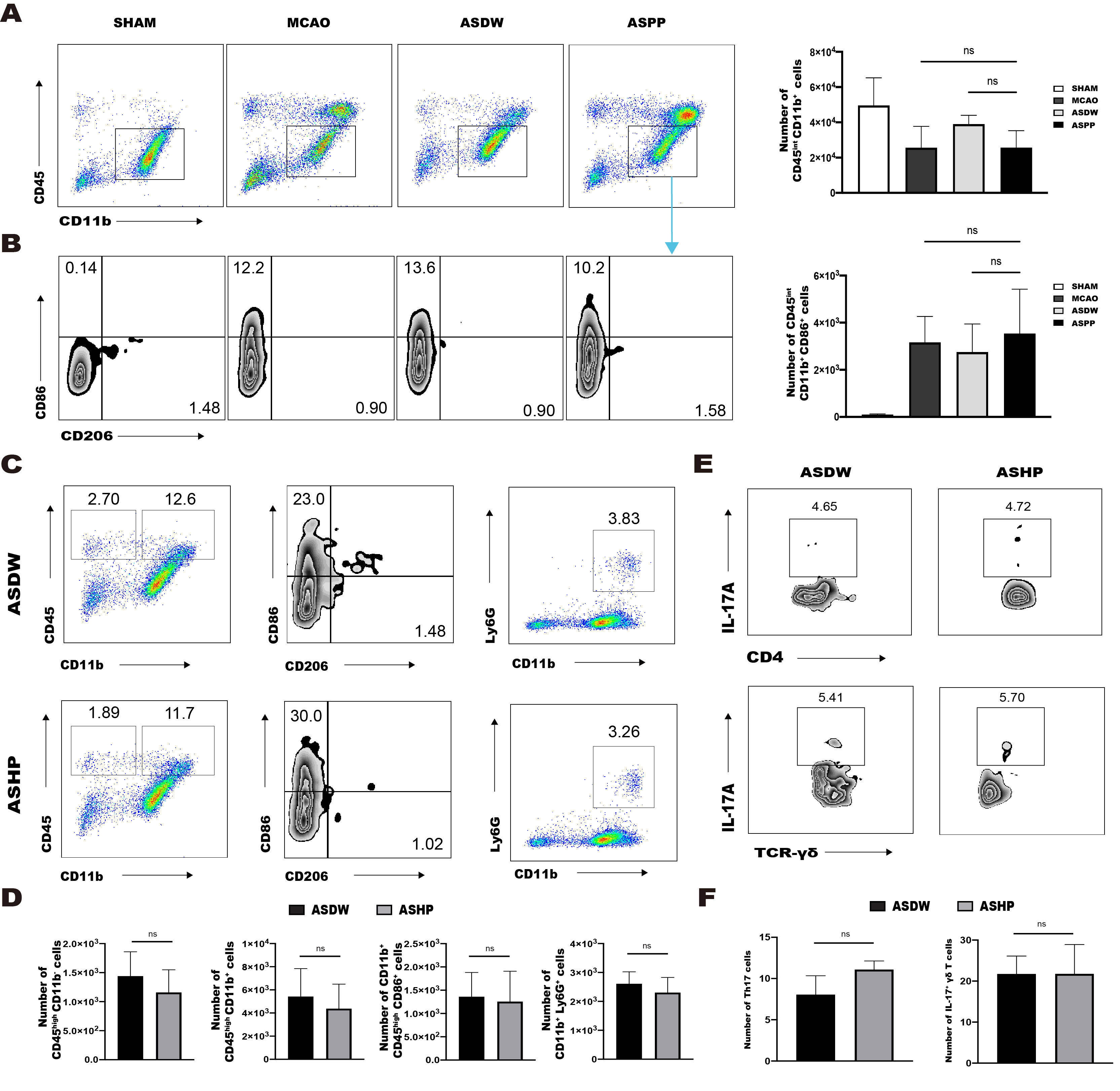

Supplement: Supplementary Figure 3 — Flow cytometry analysis of brain immune cells. (A) Gavage of salivary microbiota of periodontitis patients does not significantly alter the number of CD45intCD11b+cells in the ischemic brain 1 day after ischemic stroke. Left, representative flow cytometry analysis of CD45intCD11b+ cells. Right, quantification of CD45intCD11b+ cells. n = 6:5:6:6. (B) Gavage of salivary microbiota of periodontitis patients does not significantly alter the number of CD45intCD11b+CD86+ cells in the ischemic brain 1 day after ischemic stroke. Left, representative flow cytometry analysis of CD45intCD11b+CD86+ cells. Right, quantification of CD45intCD11b+CD86+ cells. n = 6:5:6:6. (C) Representative flow cytometry analysis of immune cells in ischemic brain of the ASDW and ASHP group after MCAO. (D) Quantifications of immune cells in (C). n = 6:6. (E) Representative flow cytometry analysis of Th17 cells and IL-17+ γδ T cells in the ischemic brain of the ASDW and ASHP group after MCAO. (F) Left, quantification of Th17 cells. n = 4:4 Right, quantification of IL-17+ γδ T cells. n = 5:5. Values represent mean ± SD. One-Way ANOVA was used for statistical analysis in (A,B), and Student’s t-test was used in (D,F). ns, not significant. [file Image_3.JPEG]

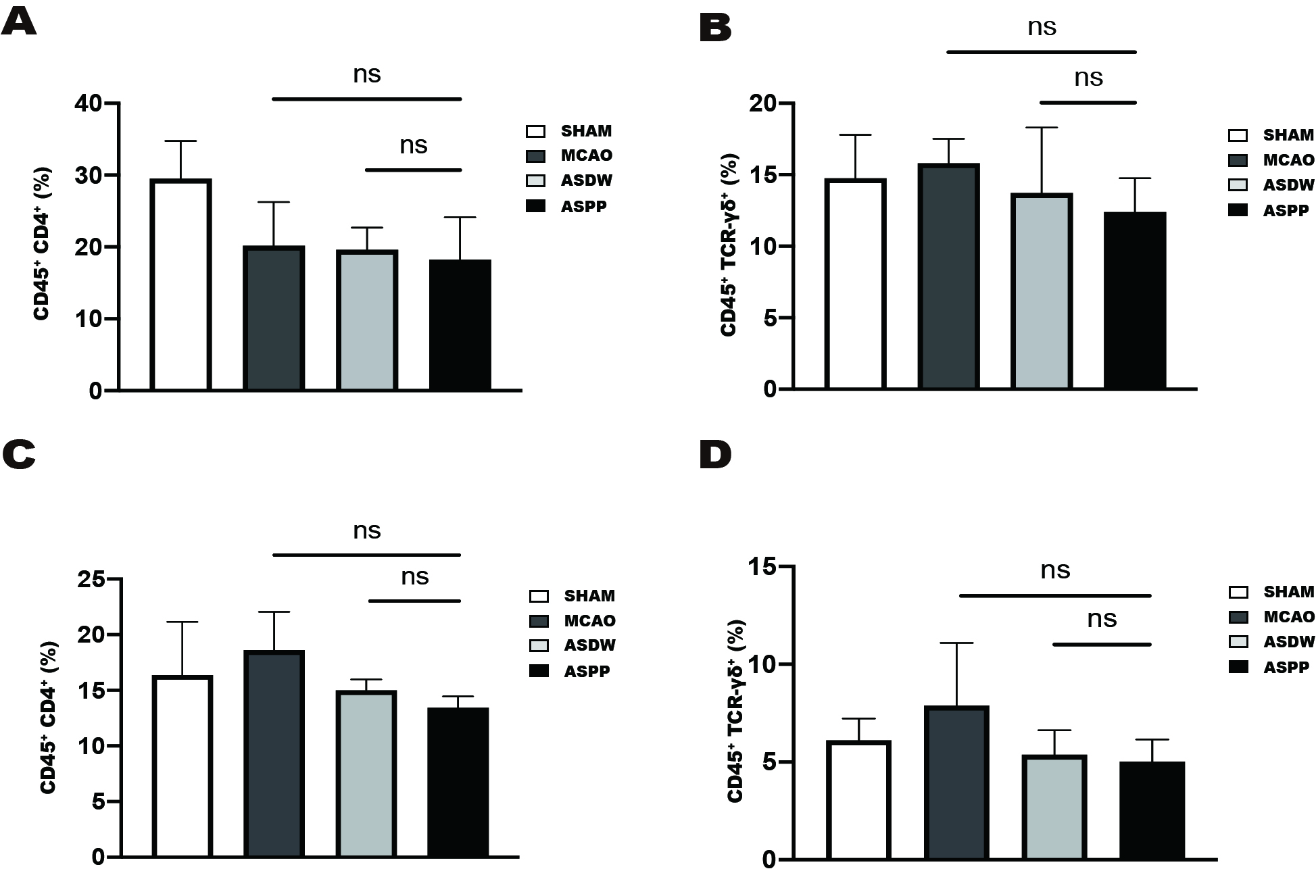

Supplement: Supplementary Figure 4 — Flow cytometry analysis of CD4+ T cells and TCR-γδ+ T cells. The ASPP group had comparable CD4+ T cells and TCR-γδ+ T cells with the ASDW and MCAO groups in the small intestine and colon. (A) Quantification of CD45+CD4+ cells in the small intestine. n = 5:5:6:6. (B) Quantification of CD45+TCR-γδ+ cells in the small intestine. n = 5:5:6:6. (C) Quantification of CD45+CD4+ cells in the colon. n = 5:5:5:5. (D) Quantification of CD45+TCR-γδ+ cells in the colon. n = 5:5:5:5. Values represent mean ± SD. One-Way ANOVA was used for statistical analysis. ns, not significant. [file Image_4.JPEG]

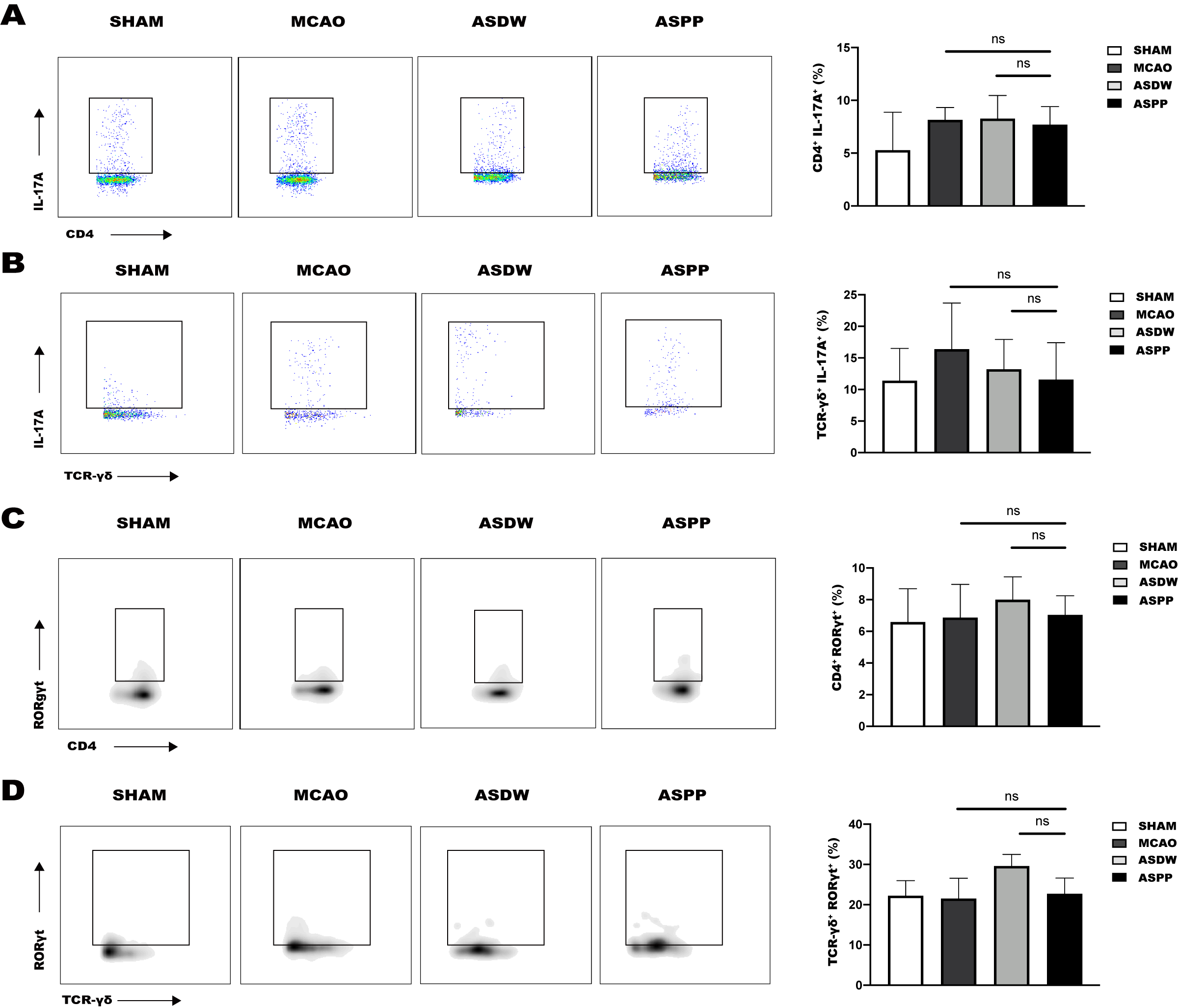

Supplement: Supplementary Figure 5 — Gavage of salivary microbiota of periodontitis patients does not affect Th17 cells or IL17+ γδ T cells in mouse colons after ischemic stroke. (A) Left, representative flow cytometry analysis of CD4+IL-17A+ cells (Th17 cells) in the colon. Right, quantification of Th17 cells. n = 5:5:6:6. (B) Left, representative flow cytometry analysis of TCR-γδ+IL-17A+ cells (IL-17+ γδ T cells) in the colon. Right, quantification of IL-17+γδ T cells. n = 5:5:5:5. (C) Left, representative flow cytometry analysis of CD4+RORγt+ T cells in the colon. Right, quantification of CD4+RORγt+ cells. n = 6:6:6:6. (D) Left, representative flow cytometry analysis of TCR-γδ+RORγt+ cells in the colon. Right, quantification TCR-γδ+RORγt+ cells. n = 5:5:5:5. Values represent mean ± SD. One-Way ANOVA was used for statistical analysis. ns, not significant. [file Image_5.PNG]

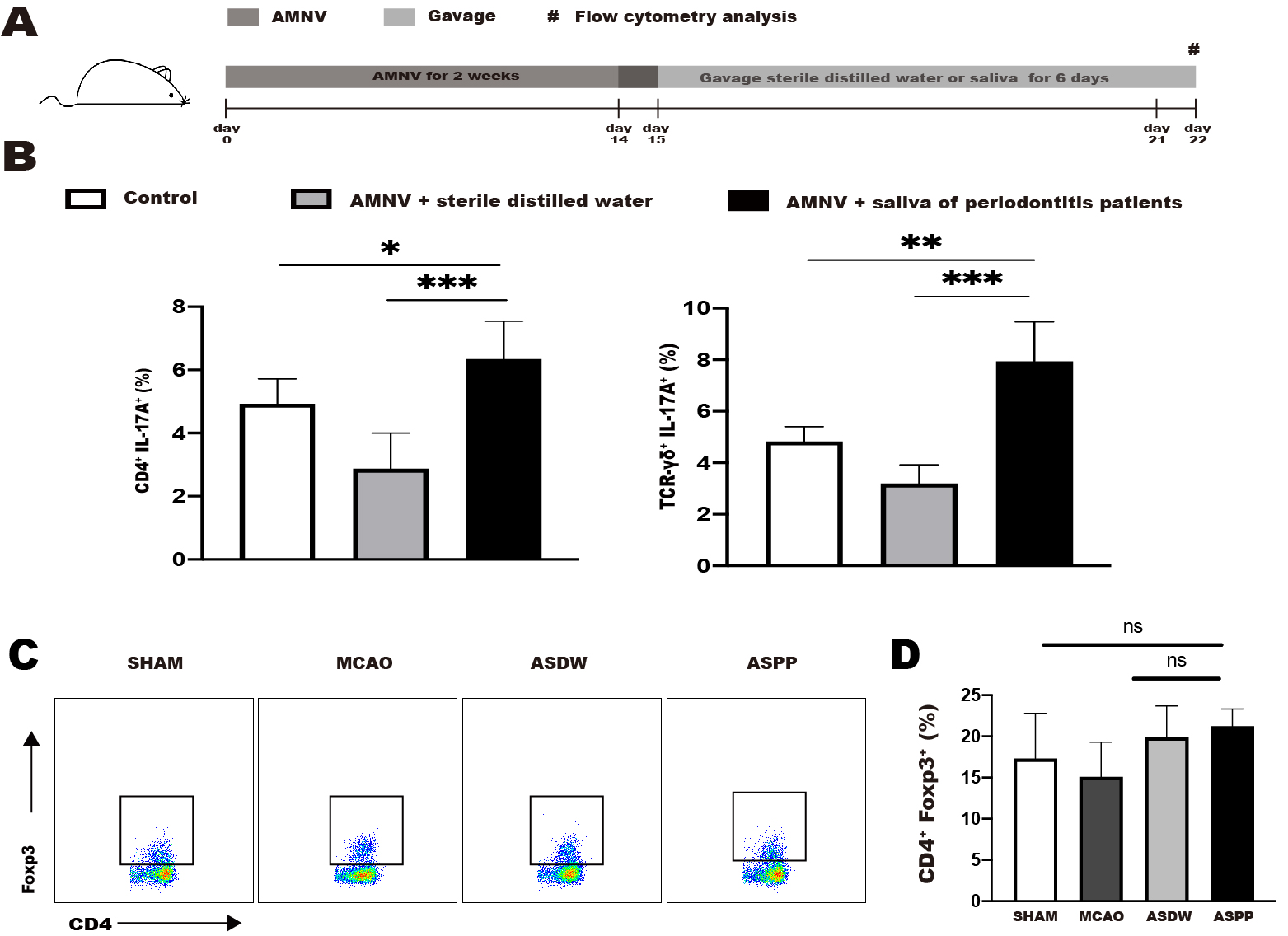

Supplement: Supplementary Figure 6 — (A) Experimental design for testing the effects of saliva of periodontitis patients without MCAO. (B) Increasing of Th17 and IL-17+ γδ T cells in the small intestine didn’t require MCAO operation. Left, quantification of Th17 cells. n = 5: 6: 6 Right, quantification of IL-17+ γδ T cells. n = 5: 5: 5. (C) Gavage of salivary microbiota of periodontitis patients does not affect CD4+Foxp3+ cells in the small intestine of mice after ischemic stroke. Representative flow cytometry analysis of CD4+Foxp3+ cells. (D) Quantification of CD4+Foxp3+ cells in (C). n = 6:6:6:6. Values represent mean ± SD. One-Way ANOVA was used for statistical analysis. ns, not significant. *p < 0.05, **p < 0.01, ***p < 0.001. [file Image_6.JPEG]

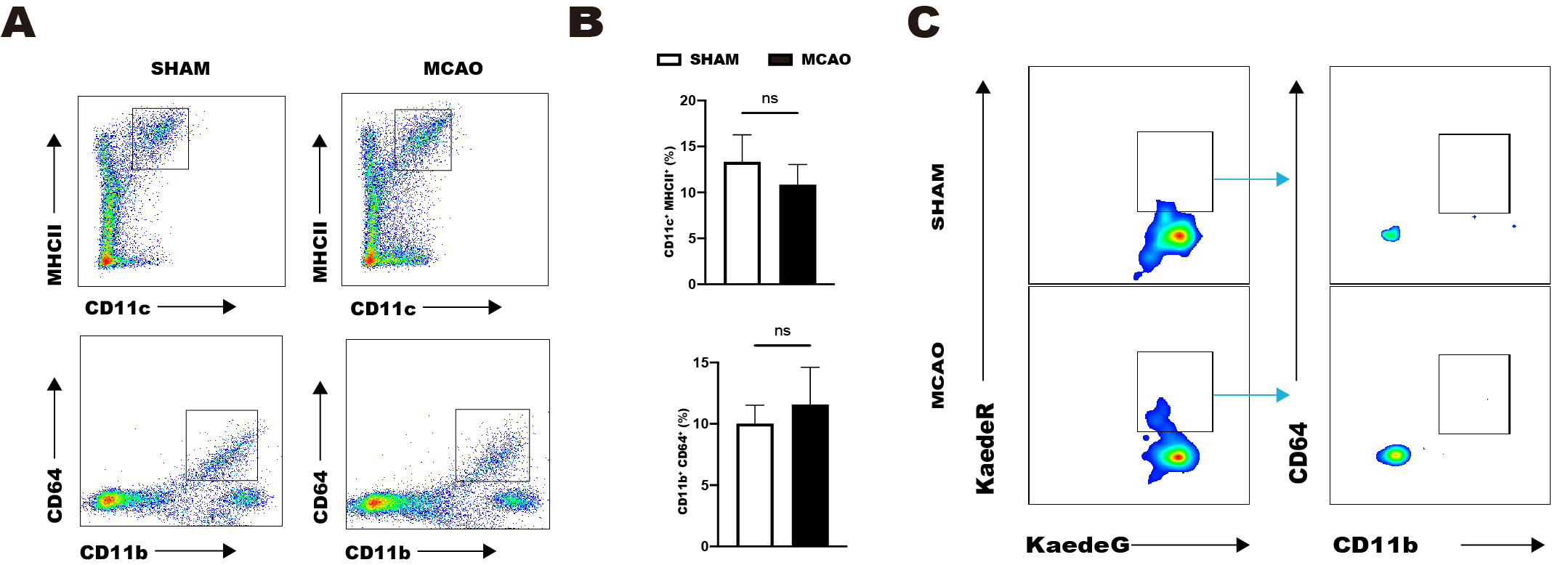

Supplement: Supplementary Figure 7 — Flow cytometry analysis of CD11c+MHCII+ cells and CD11b+CD64+ cells. MCAO does not affect dendritic cells or macrophage cells in the small intestine of mice after ischemic stroke. And macrophages do not migrate from the small intestine to the brain after ischemic stroke. (A) Representative flow cytometry analysis of CD11c+MHCII+ cells and CD11b+CD64+ cells in the small intestine. (B) Quantification of CD11c+MHCII+ cells and CD11b+CD64+ cells. n = 5: 4. (C) Representative flow cytometry analysis of KaedeR+CD11b+CD64+ macrophages in the brain of mice 1 day after MCAO or sham operation. Values represent mean ± SD. Student’s t-test was used for statistical analysis. ns, not significant. [file Image_7.JPEG]
